# Dihydroartemisinin Exerts Its Anticancer Activity through Depleting Cellular Iron via Transferrin Receptor-1

**DOI:** 10.1371/journal.pone.0042703

**Published:** 2012-08-10

**Authors:** Qian Ba, Naiyuan Zhou, Juan Duan, Tao Chen, Miao Hao, Xinying Yang, Junyang Li, Jun Yin, Ruiai Chu, Hui Wang

**Affiliations:** 1 Key Laboratory of Nutrition and Metabolism, Institute for Nutritional Sciences, Shanghai Institutes for Biological Sciences, Graduate School of Chinese Academy of Sciences, Chinese Academy of Sciences, Shanghai, China; 2 China National Center for Biotechnology Development, Beijing, China; Maastricht University Faculty of Health, Medicine, and Life Sciences, The Netherlands

## Abstract

Artemisinin and its main active metabolite dihydroartemisinin, clinically used antimalarial agents with low host toxicity, have recently shown potent anticancer activities in a variety of human cancer models. Although iron mediated oxidative damage is involved, the mechanisms underlying these activities remain unclear. In the current study, we found that dihydroartemisinin caused cellular iron depletion in time- and concentration-dependent manners. It decreased iron uptake and disturbed iron homeostasis in cancer cells, which were independent of oxidative damage. Moreover, dihydroartemisinin reduced the level of transferrin receptor-1 associated with cell membrane. The regulation of dihydroartemisinin to transferrin receptor-1 could be reversed by nystatin, a cholesterol-sequestering agent but not the inhibitor of clathrin-dependent endocytosis. Dihydroartemisinin also induced transferrin receptor-1 palmitoylation and colocalization with caveolin-1, suggesting a lipid rafts mediated internalization pathway was involved in the process. Also, nystatin reversed the influences of dihydroartemisinin on cell cycle and apoptosis related genes and the siRNA induced downregulation of transferrin receptor-1 decreased the sensitivity to dihydroartemisinin efficiently in the cells. These results indicate that dihydroartemisinin can counteract cancer through regulating cell-surface transferrin receptor-1 in a non-classical endocytic pathway, which may be a new action mechanism of DHA independently of oxidative damage.

## Introduction

Artemisinin, a natural product isolated from the plant *Artemesia annua* L., is widely used as an antimalarial drug [1], [2], [3]. Recently, more and more evidences have emerged to elucidate that artemisinin and its derivatives show potent anticancer activities in a variety of human cancer cells [4], [5], [6], [7]. Although many studies have been performed, the precise mechanism of this compound is still highly controversial [7]. It is likely that artemisinin works by multiple mechanisms. A consensus opinion is that artemisinin and its derivatives exert their antimalarial activities as well as some anticancer activities through oxidative damage. Artemisinin contains an endoperoxide bridge, which is cleaved in Fenton reaction mediated by iron and produces free-radical reactive oxygen species (ROS) [8], [9]. However, oxidative damage alone is not sufficient to explain all of the anticancer activities of artemisinin [8], [10]. It is reported that DHA can activate p38 MAPK pathway independently of ROS [10]. Another study found that in some cell lines including MCF7, iron addition did not enhance but reduce the cytotoxicity of artesunate markedly [11]. These observations are inconsistent with the previous understanding. Though some reports and our previous work have illustrated that several cellular process and pathways including cell cycle, apoptosis and invasion, etc. contribute to the anticancer activities of artemisinin [4], [5], [8], the ROS-independent mechanisms of artemisinin and its derivatives remain to be elucidated.

Iron is an essential nutrient of cells and iron homeostasis is sophisticated controlled [12]. Transferrin receptor-1 (TfR1, also known as CD71), a type II transmembrane protein, plays important roles in the cellular iron uptake and iron metabolism [13]. Most iron is delivered into cells through a TfR1 mediated endocytic pathway. Cell-surface TfR1 can bind diferric transferrin (Tf) with high affinity and the Tf-TfR1 complex is internalized through clathrin-dependent endocytosis [14], [15], [16]. In cells, TfR1 expression is regulated by intracellular iron primarily at the post-transcriptional level. There are five iron-responsive elements (IRE) in the 3′-UTR of TfR1 transcript, which is important for the breakdown of mRNA. Under low iron conditions, iron regulatory proteins (IRP) bind to the IREs and enhance the stability of mRNA, resulting in an increase of TfR1 expression [14], [17]. In cancers, the expression of TfR1 is highly elevated compared to normal tissues, which helps absorb more iron [17], [18], [19]. Cancer cells require more iron to keep rapid proliferation. And the possibility of depriving excess iron for cancer treatment, such as different kinds of iron chelators, is under investigation [19].

Here, we found that dihydroartemisinin (DHA), the main active metabolite of artemisinin derivatives, downregulated cell-surface TfR1 level through an unexpected endocytic pathway, leading to the decline of TfR1 mediated iron uptake and deficiency of cellular iron stores. This action of DHA seemed to have no relevance to oxidative damage since NAC, a kind of antioxidant, had little reversal effect. These results provide new insights into the ROS-independent anticancer mechanism of DHA and reveal a non-classical endocytic pathway of TfR1 that can be regulated by small-molecular compounds.

## Materials and Methods

### Compounds and Reagents

DHA was a kind gift of Yiwu Golden Fine Chemical Co. Ltd. Chlorpromazine hydrochloride (CPZ) was purchased from Wako Pure Chemical Industries (Saitama, Japan). Nystatin, N-Acetyl-L-cysteine (NAC), Thiazolyl Blue Tetrazolium Bromide (MTT), Deferoxamine Mesylate (DFO), ferric ammonium citrate (FAC), N-ethylmaleimide (NEM), hydroxylamine (HA) and other chemicals used in this study were purchased from Sigma-Aldrich, Inc. (St. Louis, MO).

### Cell Culture and siRNA Transfection

Human hepatoma cell line HepG2 and breast cancer cell line MCF7 originally from the ATCC were cultured in DMEM medium supplemented with 10% fetal bovine serum, 100 µg/ml penicillin and streptomycin. The siRNA transfection was performed using HiPerFect (Qiagen, Hilden, Germany) according to the manufacturer’s protocol. Stealth RNAi™ siRNA were purchased from Invitrogen (Carlsbad, CA). The sequences were as follows: control siRNA: UUC UCC GAA CGU GUC ACG UTT, ACG UGA CAC GUU CGG AGA ATT; TfR1 siRNA: UGU UAU CGCCAU CUA CUU GCC GAG C, GCU CGG CAA GUA GAU GGC GAU AAC A.

### Cellular Iron Measurement

Cells were treated with different concentrations of DHA for 24 hours. Then cells were harvested and washed with PBS three times and the relative iron content was measured as described previously [20]. Briefly, incubated the cell pellets in acid solution (3 M hydrochloric acid and 0.61 M trichloroacetic acid) for 48 hours at 65°C and vortexed for 30 min every 24 hours during the digestion. Spun down and collected the supernatant. Each sample (50 µl) was mixed with 200 µl chromagen stock: saturated sodium acetate: pure water (1∶5:5, v/v/v; chromagen stock: 1.86 mM bathophenanthroline sulfonte and 143 mM thioglycollic acid) for 10 min at room temperature. The relative iron content was determined by the absorbance at 535 nm using a SpectraMax 190 microplate reader (Molecular Devices, Sunnyvale, CA) and was normalized to the total protein concentration.

### Scanning Transmission X-ray Microscopy (STXM) Spectromicroscopic Analysis

The spatial distribution of iron in cells was determined by STXM analysis carried out at beamline 08U1-A in the Shanghai Synchrotron Radiation Facility (SSRF) as described previously [20]. HepG2 cells were treated with DHA (25 µM) for 24 hours, and then fixed in 70% ethanol overnight. The sample positioned on a high resolution X–Y–Z–θ stage was analyzed by the detector. The X-ray beam energy was scanned around the absorption K-edge of iron, between 708.5 and 702 eV with 33 steps (0.2 eV step), and then 708.5 and 707.5 eV were selected for iron standard analysis. Finally, the image was transferred to a computer for data acquisition and analysis.

### Western Blotting

Western blot analysis was performed as described previously [20]. The primary antibody against human HIF1α was obtained from BD PharMingen (San Jose, CA), anti-p53 (AB-6) was obtained from Calbiochem (San Diego, CA), anti-human Ferritin (H-chain) was obtained from Alpha Diagnostic Intl (San Antonio, TX), and anti-human β-actin (AC-74) was obtained from Sigma-Aldrich, Inc. All other antibodies together with all secondary antibodies (anti-mouse, anti-goat and anti-rabbit immunoglobulin G) were purchased from Santa Cruz Biotechnology, Inc. (Santa Cruz, CA).

### Quantitative RT-PCR Analysis

Total RNA was isolated using the TRIzol reagent (Invitrogen) and was reverse transcribed by PrimeScript™ RT reagent Kit (Takara, Otsu, Japan). Quantitative RT-PCR was performed using CFX96™ Real-Time PCR Detection System (Bio-Rad, Hercules, CA). All samples were normalized to β-actin mRNA levels. Furthermore, a dissociation curve was obtained to verify the specificity of the amplification. Primer sequences used for PCR were as follows: DMT1-F: CAG ATG ACA GTG TTT CTG GA, DMT1-R: GCA ATG GCT GAG CCA ATG AC; Steap3-F: GGC TGC TCA GCT TCT TCT G, Steap3-R: GCC AAG ACC TGC TTG ACT G; Fpn1-F: AAC CGC CAG AGA GGA TGC T, Fpn1-R: CCA AGG TAG AGA AGG AAT TTT GCA; p21-F: GAC TCT CAG GGT CGA AAA CGG, p21-R: GCG GAT TAG GGC TTC CTC TT; TfR1-F: GTC GCT GGT CAG TTC GTG ATT, TfR1-R: AGC AGT TGG CTG TTG TAC CTC TC; β-actin-F: GGC GGC ACC ACC ATG TAC CCT, β-actin-R: AGG GGC CGG ACT CGT CAT ACT.

### Cell Surface TfR1 Assay

To determine the membrane-associated TfR1 level, cells were collected, washed with PBS and resuspended in 100 µl PBS. Then 20 µl anti-human TfR1 antibody conjugated with PE (BioLegend, San Diego, CA) was added and incubated for 30 min in the dark. After removed the antibody by centrifugation and washed twice, the cells were resuspended in PBS and the membrane-associated TfR1 level was determined by measuring the mean fluorescence intensity of PE positive cells using a FACSAria flow cytometer (Becton Dickinson, Franklin Lakes, NJ). Cells without antibody incubations and cells incubated with PE conjugated mouse IgG1, κ Isotype Ctrl (BioLegend) were used as the negative control and isotype control respectively.

### Transferrin Uptake Assay

The level of transferrin uptake was determined using flow cytometry as described previously [20] . After treatment, cells were cultured in serum-free medium for 30 min to remove any residual transferrin and then were exposed to 50 µg/ml of transferrin conjugated with Alexa Fluor 633 (Invitrogen) at 37°C for different times. Endocytosis of transferrin was stopped by chilling cells on ice. Then the cells were washed with ice-cold PBS to remove external transferrin, followed by washing with pre-chilled harsh acid (0.2 M acetic acid in 0.2 M NaCl) to remove bound transferrin. After another wash with PBS, the cells were harvested and the mean fluorescence intensity of internalized transferrin in the cell population was measured by a FACSAria flow cytometer. Data are normalized to the mean fluorescence in control cells.

### Palmitoylation Assay

Palmitoylation assay was performed using the fatty acyl exchange chemistry method. Cells were harvested and endogenous TfR1 was immunoprecipitated with protein G sepharose beads (GE Healthcare, Freiburg, Germany). After washes, the beads were incubated overnight with 50 mM NEM at 4°C to block the free sulfhydryl groups followed by three times washes. Then the beads were incubated with 1 M HA at room temperature for 2 hours to cleave the Cys-palmitoyl thioester linkages. After another three times washes, the beads were incubated with 40 µM Btn-BMCC (Pierce, Rockford, IL) in dark for 2 hours to label the depalmitoylated cysteines with biotin. Biotin labeled TfR1 level was determined by immunoblotting using streptavidin-HRP antibody (Pierce), while immunoblotting with TfR1 antibody was used as the loading control. Samples without incubation with HA or Btn-BMCC were used as the negative control and NEM-nonincubated samples were used as the positive control.

**Figure 1:**
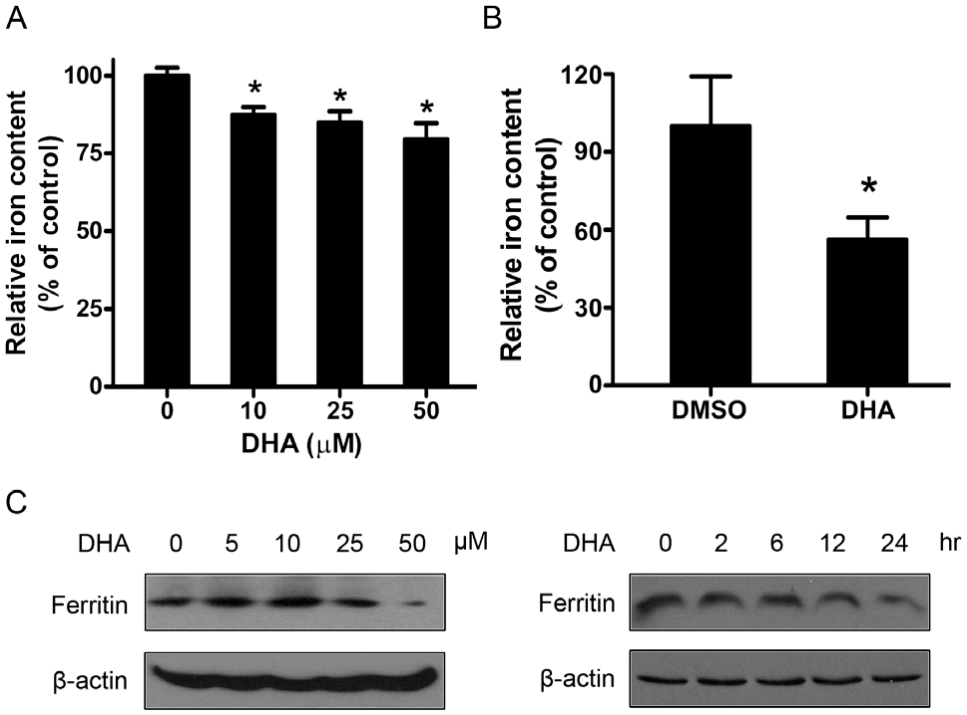
DHA reduced the iron content in cells. (A) MCF7 cells were treated with different concentrations of DHA for 24 hours. Cells were harvested and digested, and the relative iron content was determined as described in Materials and Methods. *, *P*<0.05 compared with control cells. Data are represented as mean ±SD of three different experiments. (B) HepG2 cells were treated with DMSO or 25 µM DHA for 24 hours and the cellular iron level was analyzed with STXM. *, *P*<0.05. Data are represented as mean ±SD of two different experiments. (C) HepG2 cells were treated with different concentrations of DHA for 24 hours, or treated with 25 µM DHA for different times. Cell lysates were immunoblotted to detect ferritin H-chain levels.

### Confocal Microscopy

HEK293 cells transfected with pEGFP-TfR1 (kindly provided by Dr. Hiroshi Ohno at Research Center for Allergy and Immunology, RIKEN) were grown on glass coverslips and treated with DMSO or 25 µM DHA for 24 hours. After attachment, cells were fixed with 4% paraformaldehyde in PBS for 10 min, and permeabilized with 0.1% Triton X-100 in PBS for 10 min. After wash with PBS, the coverslips were blocked with 3% BSA and incubated with primary antibody against caveolin-1 (1∶50; Santa Cruz) and then with Alexa Fluor 555-conjugated donkey anti-rabbit IgG (1∶1000, Invitrogen). After three times washes, the coverslips were mounted with ProLong® Gold antifade reagent (Invitrogen) and sealed with nail polish. Confocal images were captured with LSM510 confocal microscope (Zeiss, Jena, Germany).

**Figure 2:**
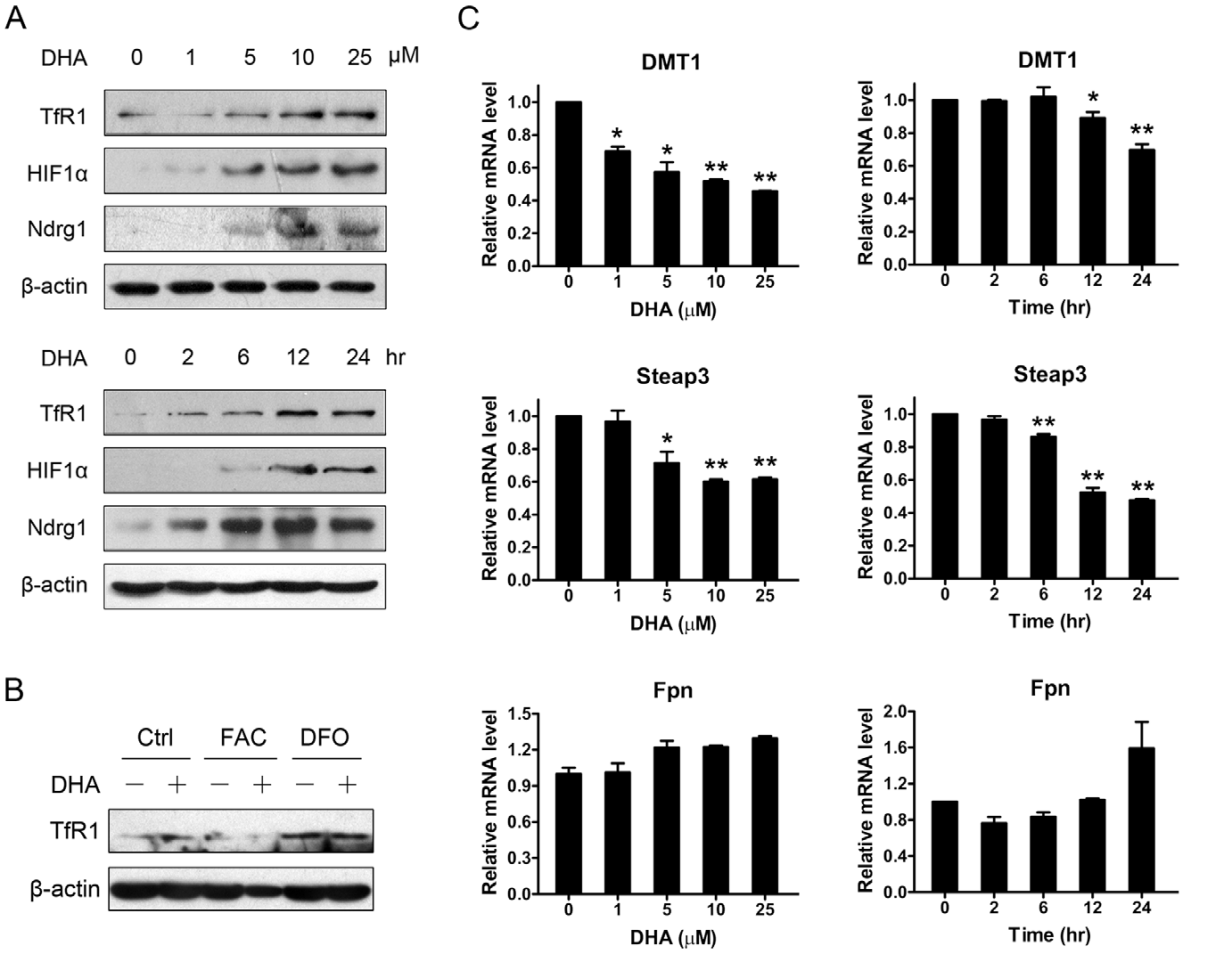
DHA caused iron depletion and weakened iron uptake pathway. (A) HepG2 cells treated with different concentrations of DHA for 24 hours or with 25 µM DHA for different times were harvested. Total proteins were extracted and western blotting was performed. (B) HepG2 cells were pretreated with DFO (250 µM) or FAC (100 µg/ml) or left untreated for 30 min and then DHA (25 µM) were added to further treatment. After 24 hours, cell lysates were prepared and immunoblotted. (C) HepG2 cells were treated as (A) and harvested to extract total RNA. The mRNA levels of endogenous DMT1, Steap3 and Fpn1 were determined by quantitative RT-PCR. *, *P*<0.05; **, *P*<0.01 compared with control cells. Data are represented as mean ±SD of three different experiments.

### Cell Viability Assay

Cell viability was determined via the MTT assay as described previously [4], [5]. Briefly, MTT solution (5 mg/ml in PBS) was added to the cells and the resultant formazan crystals were dissolved in DMSO. Absorbance at 570 nm was recorded using a SpectraMax 190 microplate reader.

**Figure 3:**
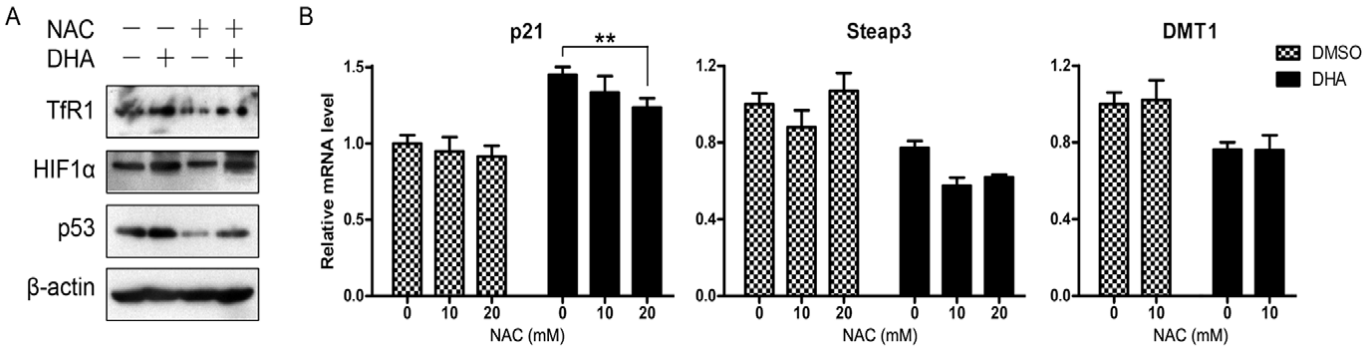
DHA induced disturbance of iron homeostasis could not be reversed by NAC. (A) MCF7 cells were pretreated with 20 mM NAC or left untreated for 30 min and then DHA (25 µM) were added to further treatment. After 24 hours, cell lysates were prepared and immunoblotted. (B) HepG2 cells were pretreated with NAC or not and further incubated with 25 µM DHA for 24 hours. Quantitative RT-PCR was performed to detect the mRNA level. **, *P*<0.01. Data are represented as mean ±SD of three different experiments.

### Statistical Analysis

The statistical significance of differences was examined using the Student’s *t*-test. Differences between groups were considered significant when *P*<0.05.

**Figure 4:**
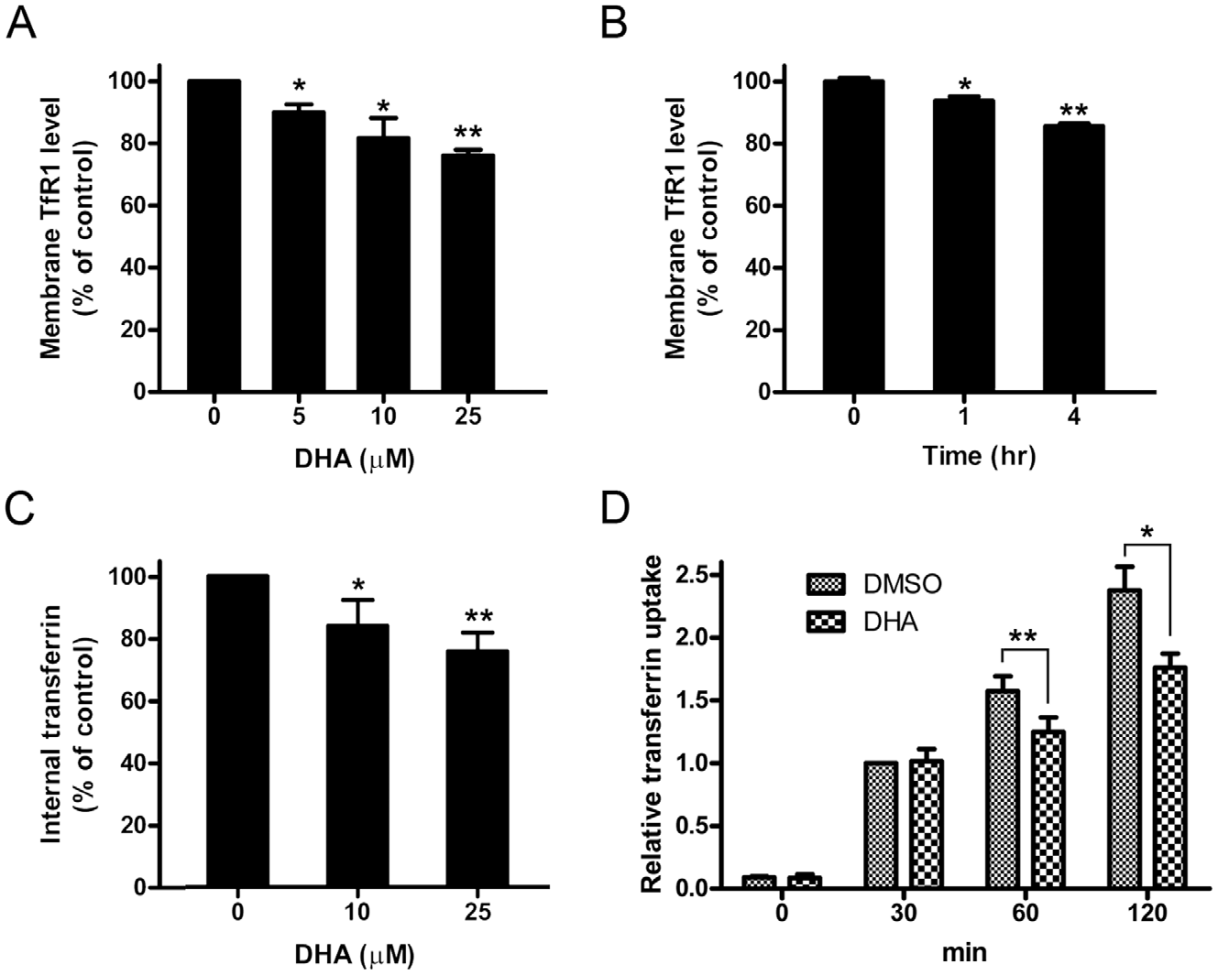
DHA decreased cell-surface TfR1 and inhibited Tf uptake. (A and B) After treated with DHA, HepG2 cells were harvested and incubated with PE-conjugated TfR1 antibody for 30 min and then subjected to flow cytometric analysis to determine membrane-associated TfR1 level. *, *P*<0.05; **, *P*<0.01 compared with control cells. Data are represented as mean ±SD of three different experiments. (C) HepG2 cells were treated with different concentrations of DHA for 24 hours and then incubated with Alexa fluor 633-conjugated transferrin for 2 hours. Harvested cells were subjected to flow cytometric analysis for internalized transferrin. *, *P*<0.05; **, *P*<0.01 compared with control cells. Data are represented as mean ±SD of three different experiments. (D) HepG2 treated with DMSO or 25 µM DHA for 24 hours were incubated with Alexa fluor 633-conjugated transferrin for the time indicated. Cells were harvested and subjected to flow cytometric analysis. *, *P*<0.05; **, *P*<0.01. Data are represented as mean ± SEM of three different experiments.

## Results

### DHA Downregulated the Cellular Iron Content

Although DHA cytotoxicity is reported as iron mediated oxidative damage, the impact of DHA on iron metabolism remains obscure. To investigate the overall effect of DHA on cellular iron metabolism, we determined the relative iron content in cells and found that DHA could reduce cellular iron stores. As shown in Fig. 1A, with the increase of DHA concentration, the total iron content in MCF7 cells reduced gradually. The STXM spectromicroscopic analysis also showed that DHA treated HepG2 cells contained less iron than control cells (Fig. 1B). Consistently, DHA decreased the protein level of ferritin, which could be a primary indicator of cellular iron stores, in concentration- and time-dependent manners in HepG2 cells (Fig. 1C). These results suggest that DHA can downregulate the total cellular iron content.

**Figure 5:**
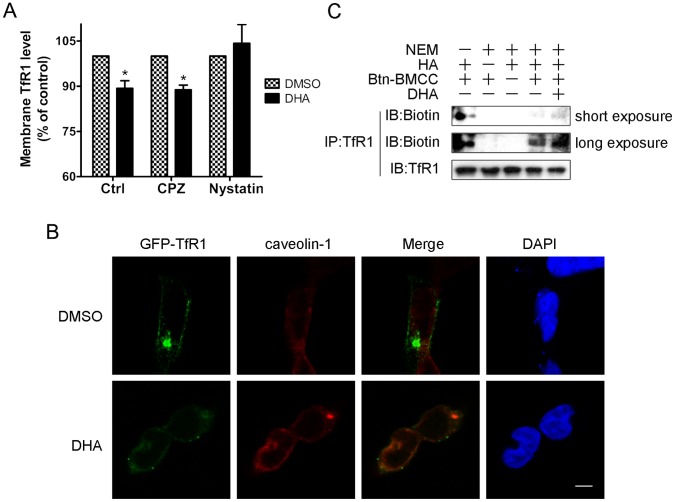
DHA induced TfR1 internalization in a lipid rafts/caveolae mediated way. (A) After pretreatment with CPZ (20 µM) or nystatin (25 µg/ml) or left untreated for 30 min, HepG2 cells were incubated with 25 µM DHA for another 4 hours. Cells were harvested and the membrane-associated TfR1 was determined by flow cytometric analysis. *, *P*<0.05 compared with DMSO-treated cells. Data are represented as mean ±SD of two different experiments. (B) HEK293 cells expressed GFP-TfR1 were treated with DMSO or 25 µM DHA for 24 hours and subjected to confocal microscope analysis. Scale bar, 5 µm. (C) HepG2 cells were treated with DMSO or 25 µM DHA for 24 hours and the endogenous TfR1 protein was immunoprecipitated to perform the palmitoylation assay as described in Materials and Methods.

### DHA Caused Cellular Iron Deficiency

We examined the expressions of three iron-responsive proteins, HIF1α, TfR1 and Ndrg1. The protein level of HIF1α is regulated by prolyl hydroxylase enzymes, which promote VHL-mediated destruction of HIF1α. And the activities of prolyl hydroxylase enzymes require oxygen and iron [21]. TfR1 mRNA could be stabilized when low content of iron exists, primarily due to iron regulatory proteins (IRPs) binding to five iron-responsive elements (IRE) in the 3′-UTR of TfR1 mRNA [12]. Ndrg1 was reported to be upregulated by some chelators, which induced iron-depletion [22]. All of these proteins as well as TfR1 mRNA were induced efficiently in concentration- and time-dependent manners by DHA treatment (Fig. 2A and S1), indicating that iron deficiency occurred in DHA treated cells. Furthermore, in iron-depleted or overloaded HepG2 cells caused by 250 µM DFO or 100 µg/ml FAC, DHA had little influence on TfR1 expression (Fig. 2B), which confirms that iron deficiency causes TfR1 induction.

**Figure 6:**
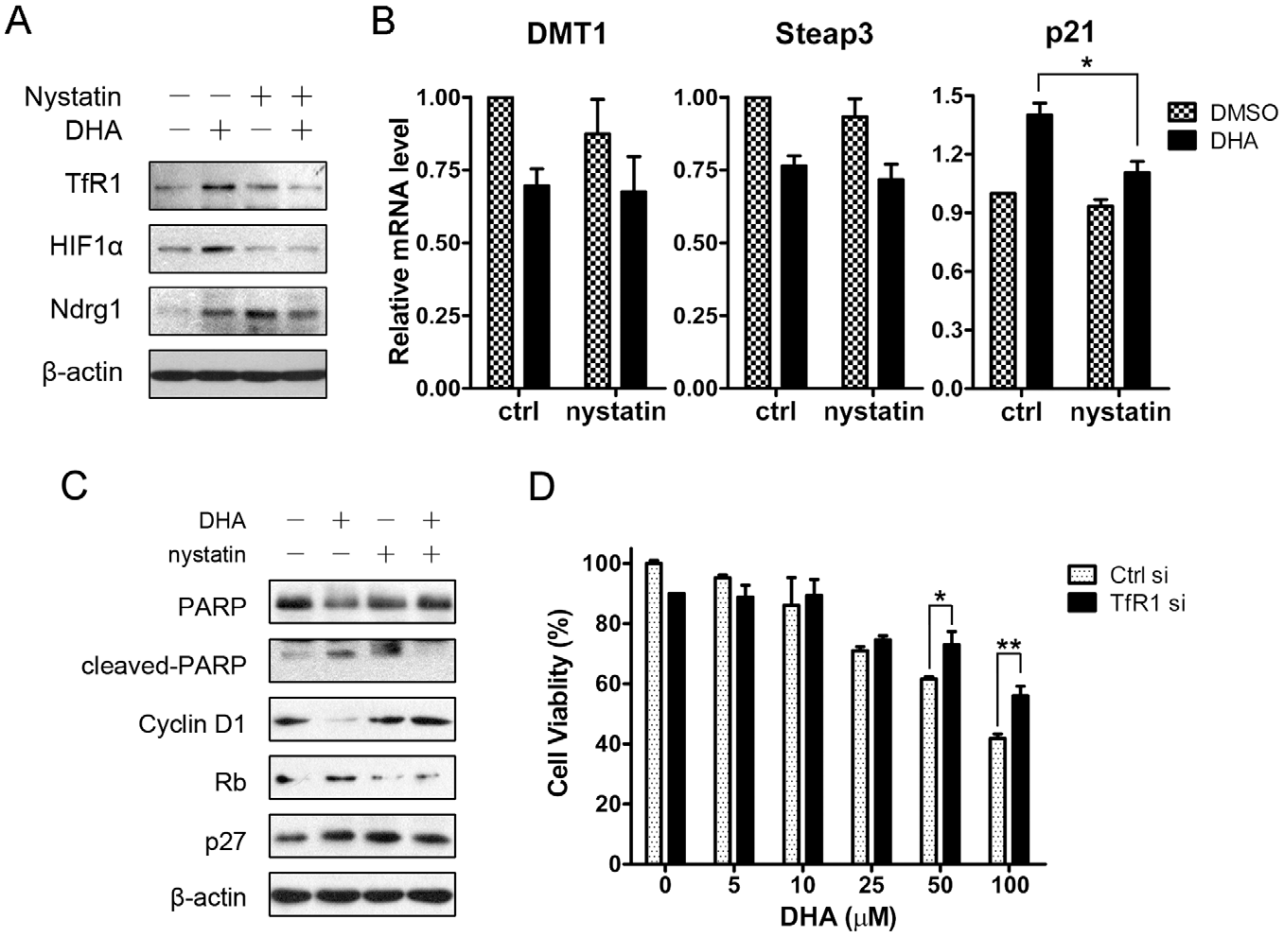
The effects of DHA were partially dependent on TfR1 internalization. (A) HepG2 cells were preincubated with 25 µg/ml nystatin or not for 30 min and treated with 25 µM DHA for another 4 hours. Cells were lysed to western blot assay. (B) HepG2 cells were preincubated with 25 µg/ml nystatin or not for 30 min and treated with 25 µM DHA for another 24 hours. Total RNA was harvested and quantitative RT-PCR was performed. *, *P*<0.05. Data are represented as mean ± SEM of more than three different experiments. (C) HepG2 cells were treated as (B) and lysed to western blot assay. (D) After transfected with 30 nM siRNA for 24 hours, HepG2 cells were treated with DHA for another 24 hours. Cell viability was determined via MTT assay. *, *P*<0.05; **, *P*<0.01. Data are represented as mean ±SD of three different experiments.

### DHA Weakened the Expressions of Iron Uptake Genes

Iron deficiency could result from decreased cellular iron uptake and/or increased iron exportation. To better understand how DHA causes iron deficiency, we examined the expressions of several genes involved in iron acquisition or release process. TfR1 mediated iron uptake plays important roles in iron acquisition, and this process requires some accessory proteins including Steap3 and DMT1. By binding transferrin, ferric iron was internalized through endocytosis into endosome. Under the acidic conditions inside the endosome, ferric iron was released and reduced to ferrous iron by the ferrireductase, Steap3. The reduced iron was then transported into the cytosol by DMT1 [14]. In both HepG2 and MCF7 cells, we found that DHA treatment reduced Steap3 and DMT1 expression significantly, suggesting that the process of iron acquisition was weakened by DHA (Fig. 2C and S2). Furthermore, the expression of Fpn1, by which the iron was transported out of the cells [23], [24], increased slightly after DHA treatment, although this change was not significant (Fig. 2C). These results suggest that DHA may interfere with cellular iron acquisition and iron release, which may be one reason why DHA induces iron deprivation in cells.

**Figure 7:**
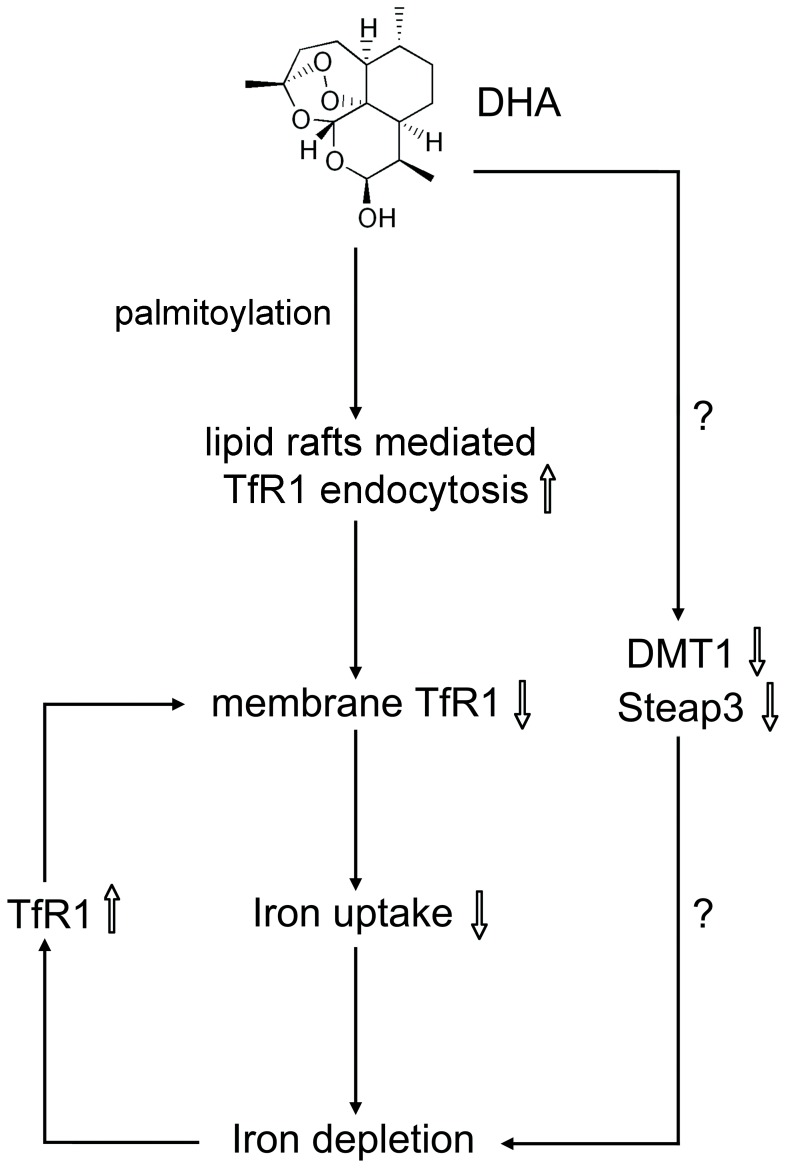
The proposed mechanisms by which DHA disrupts iron homeostasis.

### DHA Disrupted Cellular Iron Homeostasis Independent of Oxidation Damage

Considering the important action mechanism of DHA is that the cellular iron mediated endoperoxide bridge cleavage to result in free radicals and subsequent oxidative damage, we wondered whether DHA could disrupt iron homeostasis through oxidative damage. Therefore, NAC, an antioxidant, was employed to perform a rescue assay and cellular ROS production was examined to validate the antioxidative effect (data not shown). In MCF7 cells, NAC pretreatment significantly decreased p53 levels both in DMSO and DHA treated groups (Fig. 3A). However, NAC could not protect cells from DHA-induced damage completely. After cells were pretreated with NAC, DHA could still induce TfR1 and p53 (Fig. 3A). Similarly, though NAC pretreatment partially reversed DHA induced p21 expression, it had little effect on the reduction of Steap3 and DMT1 caused by DHA (Fig. 3B). These findings suggest that DHA disturbs iron homeostasis through an alternative mechanism which may be independent of oxidation damage.

### DHA Decreased the Levels of Cell-surface TfR1 and Cellular Tf Uptake

To explore the mechanisms of how DHA causes cellular iron depletion, we determined the effect of DHA on the level of cell-membrane associated TfR1 and cellular Tf uptake. We found that after DHA treatment the cell-surface TfR1 level was reduced significantly in a concentration-dependent manner (Fig. 4A). To eliminate the influence of cell viability, short-term treatments within 4 hours were performed. Our results showed that DHA decreased membrane-associated TfR1 even in the first hour of treatment (Fig. 4B). During that time, DHA had little effect on cell viability, suggesting this phenomenon was not due to the side-effects of cytotoxicity. Consistently with cell-surface TfR1, the total internalized Tf (Fig. 4C) as well as the efficiency of Tf uptake (Fig. 4D) were decreased by DHA treatment.

### DHA Induced TfR1 Internalization in a Lipid Rafts/caveolae Mediated Way

Interestingly, though DHA increased total cellular TfR1 expression, the cell surface TfR1 level was reduced after DHA treatment. This made us speculate that DHA treatment may induce abnormal endocytosis of TfR1. Thus, we utilized two drugs to inhibit the process of endocytosis: clathrin-dependent endocytosis inhibitor chlorpromazine (CPZ) and cholesterol-sequestering agent nystatin, which inhibits lipid rafts/caveolae mediated endocytosis [25], [26], [27]. Surprisingly, CPZ had little effect on DHA caused cell-surface TfR1 reduction. In contrast, in nystatin pretreated cells, the decrease of cell-surface TfR1 was almost completely reversed (Fig. 5A). Furthermore, we found that in control cells GFP-TfR1 showed little colocalization with caveolin-1, however, DHA treatment increased the colocalization of GFP-TfR1 and caveolin-1 significantly (Fig. 5B). These results suggest that DHA induces TfR1 endocytosis in a lipid rafts/caveolae mediated nystatin-sensitive manner, which is distinct from the typical way that TfR1 is internalized under normal physiological conditions. As protein palmitoylation represents an important mechanism governing the dynamic protein subcellular localization including translocation to lipid rafts, we determined TfR1 palmitoylation level using fatty acyl exchange chemistry method. As shown in Fig. 5C, biotin labeled TfR1 was shown strong in NEM omitted group and undetectable in HA or Btn-BMCC omitted groups. These results confirm the reliability of our method. Interestingly, we found that DHA enhanced the palmitoylation of TfR1 in HepG2 cells (Fig. 5C), which could at least partially explain the mechanism of DHA induced TfR1 endocytosis.

### The Effects of DHA were Partially Dependent on TfR1 Internalization

To further investigate the role of DHA induced TfR1 internalization, we used nystatin to block the lipid rafts/caveolae mediated endocytosis. And we found that in cells preincubated with nystatin, DHA was incapacitated from induction of cellular TfR1, HIF1α and Ndrg1 expression (Fig. 6A). These results demonstrate the important roles of TfR1 internalization in DHA mediated iron depletion. However, nystatin pretreatment could not protect Steap3 and DMT1 from DHA-induced reduction (Fig. 6B), which suggests that DHA downregulates these genes in other ways. Furthermore, we found that in nystatin pretreated cells, the effects of DHA on cell cycle and apoptosis related genes, including p21, PARP, cyclin D1, Rb, p27, were reversed (Fig. 6B and C), suggesting that DHA may induce cell cycle arrest and trigger apoptosis partially through TfR1 internalization. To validate the role of TfR1 in the cytotoxicity of DHA, we inhibited TfR1 expression using specific siRNA (Fig. S3). As expected, after endogenous TfR1 was knocked down, the cytotoxicity of DHA was efficiently reduced (Fig. 6D).

## Discussion

Our previous work has shown that DHA is the most active compound among artemisinin and its three derivatives to inhibit liver and ovarian cancer growth [4], [5]. In the present study, we found that DHA could disrupt cellular iron homeostasis through inducing TfR1 palmitoylation and internalization to realize its cytotoxicity. Based on our observations, we speculated that DHA reduced cellular iron content and caused iron depletion through triggering TfR1 endocytosis in a lipid rafts/caveolae mediated pathway and subsequently disrupting iron uptake (Fig. 7). Our reports not only characterized a new mechanism of DHA, but also revealed an unusual endocytic pathway of TfR1 that could be targeted by small-molecular compounds.

Artemisinin and its derivatives have been used as antimalarial drugs for nearly two decades [2], [9], and their potential application in cancer treatment is also promising. It has been proposed that the toxicity of artemisinin related compounds is attributed to iron-mediated ROS generation and cancer cells receive more severe damage due to the elevated iron level [7], [8], [9]. However, some activities of artemisinin cannot be explained by the model. It has been discovered that DHA activates p38 MAPK pathway independently of ROS in HL60 cells [10]. Another group reported that cancer cells showed different responses to artesunate plus iron salt. In some cell lines including MCF7, iron addition even reduced the cytotoxicity markedly [11]. These observations were contradictory to previous understanding. Kelter and colleagues found that TfR1 might be a determinant [11]. In this report, we evaluated the effects of DHA on iron homeostasis and proposed a new action mechanism independent of oxidative damage. DHA induces TfR1 abnormal endocytosis and causes cellular iron deficiency subsequently, and cells get harmed by iron deficiency. Our hypotheses can not only explain why iron addition decreased but not increased the cytotoxicity of DHA in some cells, but also explain why DHA selectively kills cancer cells: (1) cancer cells express higher level of TfR1 [17], thus show more susceptible to DHA than normal cells; (2) cancer cells need excess iron for rapid proliferation, so iron deficiency is more toxic to cancer cells than to normal cells [17], [20].

In some studies, artemisinin decreased HIF1α expression in mouse embryonic stem cell-derived embryoid bodies, Rheumatoid arthritis fibroblast-like synoviocytes, and C6 glioma cells [28], [29], [30]. However, in our report, we observed that DHA increased HIF1α expression independently of ROS. It is most likely that in different cell types and experimental settings, artemisinins exert different activities. In our system, HIF1α was induced by iron depletion. Actually, HIF1α has been reported to be activated by artemisinin in several cancer cell lines including HepG2 and MCF7 cells [31].

The process through which TfR1 mediates deferric Tf uptake is well described, and involves the clathrin dependent endocytic pathway [14], [27]. Interestingly, an alternative endocytic pathway of TfR1 mediated by lipid rafts has been discovered recently [32]. Horonchik and colleagues have found that a small-molecular compound, ferristatin, could regulate TfR1 degradation by inducing its internalization, which is sensitive to cholesterol depletion [32]. We observed that TfR1 endocytosis was reversed by nystatin, a cholesterol-sequestering agent that inhibits membrane trafficking mediated by lipid-rafts. Thus, we confirmed the non-classical endocytic pathway for TfR1 internalization, and also found that the process could be regulated by DHA. Both Horonchik’s report and ours have shown that small molecular compounds can regulate TfR1 internalization in a lipid-rafts mediated way, indicating that this unusual endocytic pathway of TfR1 might be a drug target to inhibit iron uptake. More compounds need to be studied on membrane trafficking of TfR1 to determine whether this is a common mechanism. The difference between our and Horonchik’s studies is that we found the total TfR1 expression elevated as a result of induced endocytosis, which might be due to iron depletion and the increased mRNA level of TfR1 (Fig. 7).

Besides, TfR1 was exploited to be an effective targeting molecular to deliver therapeutic agents into cancer cells [33]. Drugs conjugated to TfR1 antibody or Tf bind cell-surface TfR1 and undergo absorption through clathrin dependent endocytosis [33]. How lipid-rafts mediated membrane trafficking of TfR1 influences the TfR1-targeted drug delivery? Whether TfR1 can mediate DHA uptake through the unusual endocytosis? These possibilities remain unclear and need further investigation.

DHA was also found to decrease the expressions of DMT1 and Steap3, two major participants of iron internalization. However, the change of DMT1 and Steap3 appeared more slowly (6–12 hours) than that of cell-surface TfR1 (1 hour) and iron-responsive proteins (HIF1α, TfR1 and Ndrg1) (2 hours). So it is unlikely that DHA causes iron depletion through DMT1 and Steap3. Besides, the decrease of DMT1 and Steap3 was not reversed by nystatin, indicating that their expression changes were not mediated by TfR1 internalization. These results show that DHA may influence iron metabolism in multiple ways (Fig. 7). In addition to pumping ferrous iron into cytosol from endosome, DMT1 was found to be a major transporter of non-transferrin-bound iron (NTBI) [14]. The downregulation of DMT1 expression induced by DHA raises the possibility that DHA may inhibit Tf-bound iron as well as NTBI uptake. Furthermore, other mechanisms of DHA to disturb iron metabolism cannot yet be excluded.

In summary, we demonstrated that DHA could induce TfR1 internalization by a lipid rafts mediated way, and could also disrupt cellular iron uptake independently of oxidative damage, which might be another mechanism of DHA to counteract cancer. These results shed new lights on the actions of artemisinin and its derivatives in cancer cells. Future studies will focus on the physiological role of membrane trafficking of TfR1 in tumorigenesis and how this process impacts the efficacy of different therapeutic compounds.

## Supporting Information

Figure S1 **DHA induced TfR1 protein expression in MCF7 cells.** MCF7 cells were treated with DHA and cell lysates were immunoblotted to detect TfR1.
(TIF)

Figure S2 **DHA changed gene expressions in MCF7 cells.** MCF7 cells were treated with DHA. Total RNA was extracted and quantitative RT-PCR was performed. *, *P*<0.05; **, *P*<0.01 compared with control cells. Data are represented as mean ± SEM of three different experiments.
(TIF)

Figure S3 **The siRNA knocked down TfR1 expression significantly.** HepG2 cells were transfected with siRNA (30 nM) for 48 hr and quantitative RT-PCR was performed. **, *P*<0.01 compared with blank. Data are represented as mean ±SD of three different experiments.
(TIF)
