# Supplementary figures and images for: Dihydroartemisinin Exerts Its Anticancer Activity through Depleting Cellular Iron via Transferrin Receptor-1
Source: PLoS One. 2012 Aug 10;7(8):e42703. doi: 10.1371/journal.pone.0042703 (PMC3416848; doi:10.1371/journal.pone.0042703)

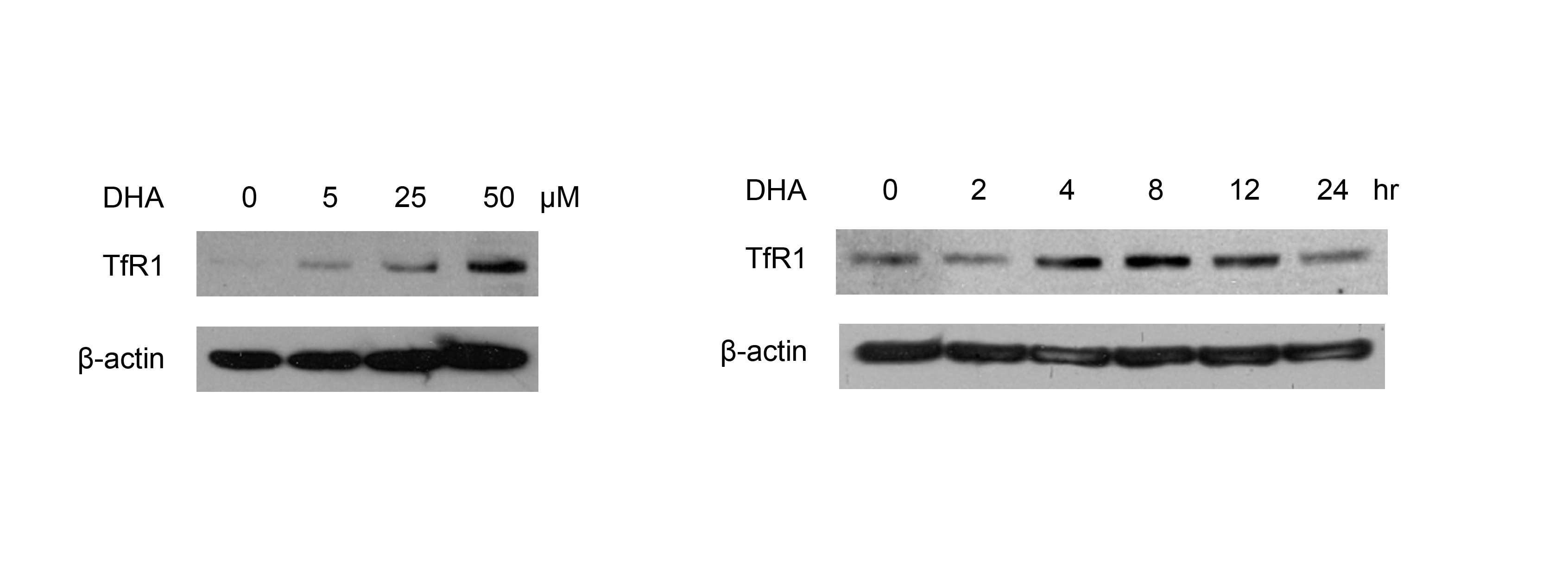

Supplement: Figure S1 — DHA induced TfR1 protein expression in MCF7 cells. MCF7 cells were treated with DHA and cell lysates were immunoblotted to detect TfR1. (TIF) [file pone.0042703.s001.tif]

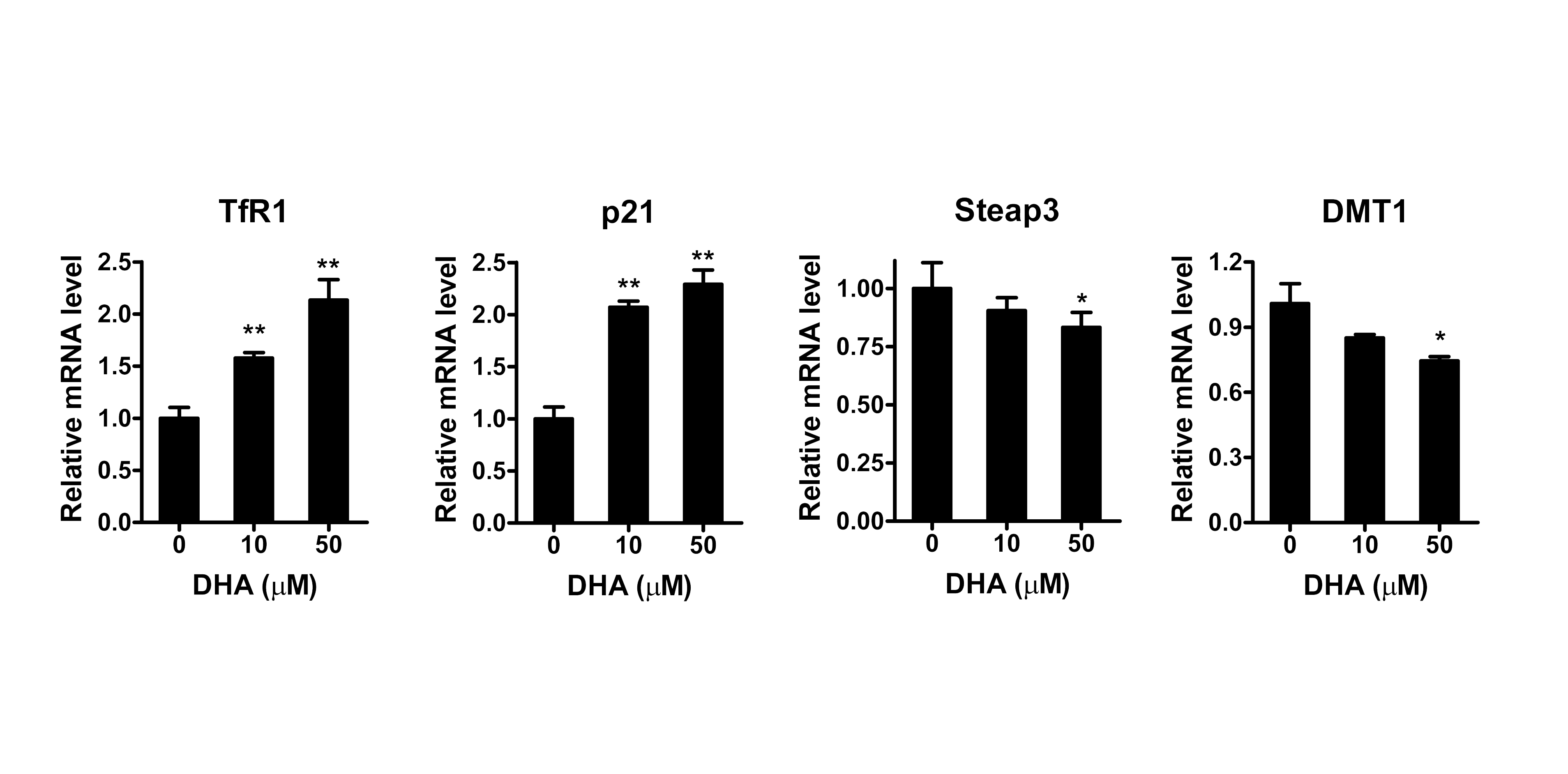

Supplement: Figure S2 — DHA changed gene expressions in MCF7 cells. MCF7 cells were treated with DHA. Total RNA was extracted and quantitative RT-PCR was performed. *, P<0.05; **, P<0.01 compared with control cells. Data are represented as mean ± SEM of three different experiments. (TIF) [file pone.0042703.s002.tif]

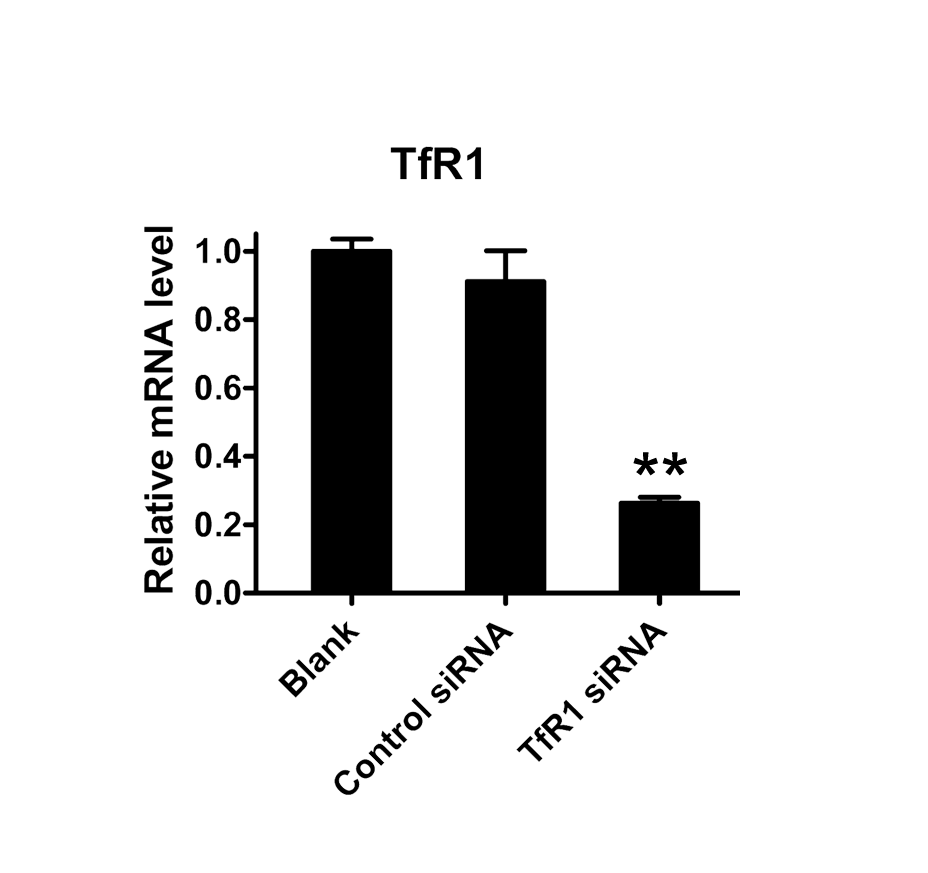

Supplement: Figure S3 — The siRNA knocked down TfR1 expression significantly. HepG2 cells were transfected with siRNA (30 nM) for 48 hr and quantitative RT-PCR was performed. **, P<0.01 compared with blank. Data are represented as mean ±SD of three different experiments. (TIF) [file pone.0042703.s003.tif]
